# Cerebral Blood Volume Analysis in Glioblastomas Using Dynamic Susceptibility Contrast-Enhanced Perfusion MRI: A Comparison of Manual and Semiautomatic Segmentation Methods

**DOI:** 10.1371/journal.pone.0069323

**Published:** 2013-08-08

**Authors:** Seung Chai Jung, Seung Hong Choi, Jeong A. Yeom, Ji-Hoon Kim, Inseon Ryoo, Soo Chin Kim, Hwaseon Shin, A. Leum Lee, Tae Jin Yun, Chul-Kee Park, Chul-Ho Sohn, Sung-Hye Park

**Affiliations:** 1 Department of Radiology, Seoul National University College of Medicine, Seoul, Korea; 2 Department of Neurosurgery, Seoul National University College of Medicine, Seoul, Korea; 3 Department of Pathology, Seoul National University College of Medicine, Seoul, Korea; University of Manchester, United Kingdom

## Abstract

**Purpose:**

To compare the reproducibilities of manual and semiautomatic segmentation method for the measurement of normalized cerebral blood volume (nCBV) using dynamic susceptibility contrast-enhanced (DSC) perfusion MR imaging in glioblastomas.

**Materials and Methods:**

Twenty-two patients (11 male, 11 female; 27 tumors) with histologically confirmed glioblastoma (WHO grade IV) were examined with conventional MR imaging and DSC imaging at 3T before surgery or biopsy. Then nCBV (means and standard deviations) in each mass was measured using two DSC MR perfusion analysis methods including manual and semiautomatic segmentation method, in which contrast-enhanced (CE)-T1WI and T2WI were used as structural imaging. Intraobserver and interobserver reproducibility were assessed according to each perfusion analysis method or each structural imaging. Interclass correlation coefficient (ICC), Bland-Altman plot, and coefficient of variation (CV) were used to evaluate reproducibility.

**Results:**

Intraobserver reproducibilities on CE-T1WI and T2WI were ICC of 0.74–0.89 and CV of 20.39–36.83% in manual segmentation method, and ICC of 0.95–0.99 and CV of 8.53–16.19% in semiautomatic segmentation method, repectively. Interobserver reproducibilites on CE-T1WI and T2WI were ICC of 0.86–0.94 and CV of 19.67–35.15% in manual segmentation method, and ICC of 0.74–1.0 and CV of 5.48–49.38% in semiautomatic segmentation method, respectively. Bland-Altman plots showed a good correlation with ICC or CV in each method. The semiautomatic segmentation method showed higher intraobserver and interobserver reproducibilities at CE-T1WI-based study than other methods.

**Conclusion:**

The best reproducibility was found using the semiautomatic segmentation method based on CE-T1WI for structural imaging in the measurement of the nCBV of glioblastomas.

## Introduction

Perfusion magnetic resonance (MR) imaging has recently become one of the most important methods for the characterization of gliomas. Dynamic susceptibility contrast-enhanced (DSC) perfusion MR imaging has been widely used clinically for perfusion MR imaging. The DSC perfusion technique indicates physiologic information about neovascularity and angiogenesis for the entire brain [1], [2]. T2- or T2*-weighted echo-planar MR sequences have been used to demonstrate dynamic changes of signal intensity during the first passage of a bolus of paramagnetic intravascular contrast agents because of the excellent temporal resolution in the entire brain [2]–[4].

The relative cerebral blood volume (rCBV) map derived from DSC MR imaging has been used for the evaluation of gliomas. The rCBV values can be calculated from the measurement of dynamic changes in signal intensity on a pixel-by-pixel basis [2]–[4]. The rCBV values correlate with glioma grading and tumor microvascular degree. High-grade gliomas are known to present higher rCBV values compared to low-grade gliomas [2], [5]–[7]. Various histogram analyses of brain tumors are possible because of the measurement of rCBV on a pixel-by-pixel basis in the entire brain [7]–[9].

Reliable and reproducible evaluation in perfusion analysis has become important in the clinical management of patients with gliomas as well as in clinical trials investigating the efficacy of anti-angiogenic agents [10]. Semi-quantitative measurement based on histogram analyses is considered to demonstrate better reproducibility than conventional region-of-interest (ROI) methods [3], [4]. However, these results still have limitations concerning the objective and reproducible measurement of rCBV. These histogram analyses, designated manual analyses here, can result in observer-dependent measurement. Semiautomatic segmentation helped to reduce variability in the analysis of tumors [11], [12]. Thus, the reproducibility in perfusion MR imaging analysis needs to be established with the conventional manual segmentation method and semiautomatic segmentation method.

In this study, we quantitatively compared two methods of segmenting glioblastomas for the measurement of rCBV from DSC perfusion MR image data: the conventional manual segmentation method and a semiautomatic segmentation method. To compare the inter- and intraobserver reliability of the two methods, two observers performed tumor segmentation twice with each method in the same subjects. Our hypotheses were that (a) the manual segmentation method would be more user dependent, i.e., have lower inter- and intraobserver reliability, and (b) the semiautomatic segmentation method would provide more reproducible measurements, i.e., have high inter- and intraobserver reliability.

## Materials and Methods

This retrospective study was approved by the institutional review board of Seoul National University Hospital, and patients' informed consent was waived.

### Patient selection

**Figure 1:**
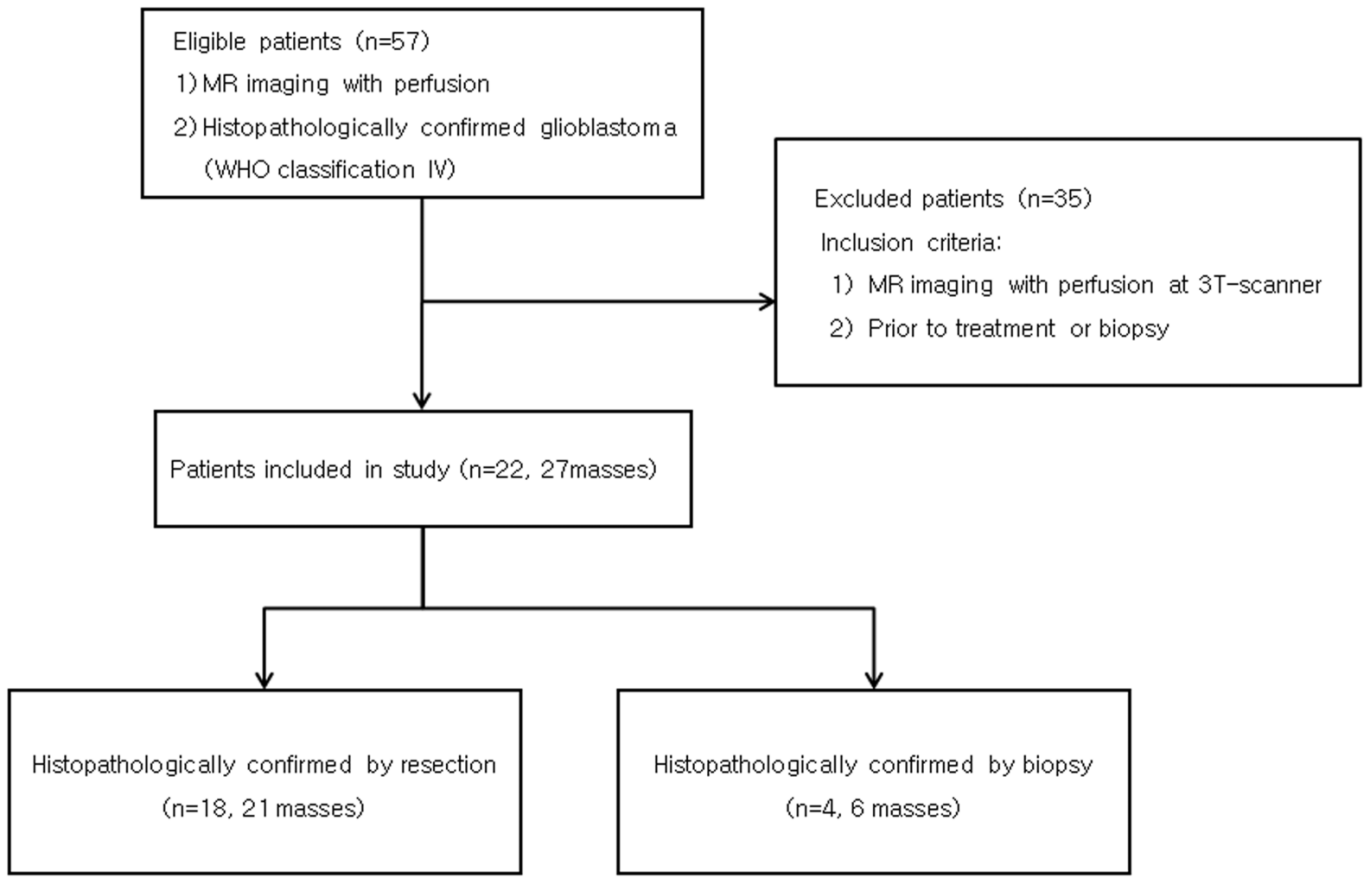
Flowchart of patient selection and inclusion criteria. MR = magnetic resonance; WHO = World Health Organization.

Between February 2010 and May 2012, 51 patients receiving initial DSC perfusion MR imaging at our institute were diagnosed with grade IV glioblastoma on the basis of the World Health Organization histopathologic criteria. Of the patients, only 22 patients were enrolled in this study. These 22 patients underwent MR imaging using a 3T-scanner prior to treatments or biopsy. The enrolled patients consisted of 11 males and 11 females (mean age, 52.5 years; age range, 20–72 years; 27 tumors, 5 patients with two masses) (Fig. 1).

### MR imaging protocol

Twenty-two patients underwent conventional MR imaging and DSC perfusion MR imaging using a 3T-scanner (Verio; Siemens Healthcare Sector, Erlangen, Germany) with a 32-channel head coil. The conventional MR imaging included T1-weighted imaging (T1WI), such as transverse spin-echo imaging, before and after contrast enhancement or multi-planar reconstructed transverse, coronal imaging with a sagittal three-dimensional magnetization prepared rapid acquisition gradient echo (3D-MPRAGE) sequence before and after contrast enhancement, and transverse T2-weighted imaging (T2WI) with turbo spin-echo sequences. Contrast-enhanced (CE) T1WI was acquired after the intravenous administration of gadobutrol (Gadovist®, Bayer Schering Pharma, Berlin, Germany) at a concentration of 0.1 mmol per kilogram (mmol/kg) of body weight. The transverse spin-echo T1-weighted imaging was obtained with the following parameters: repetition time (TR), 558 ms; echo time (TE), 9.8 ms; flip angle (FA), 70°; matrix, 384×187; field-of-view (FOV), 175×220 mm; section thickness, 5 mm; and number of excitations (NEX), 1. We obtained the 3D-MPRAGE sequences using the following parameters: TR, 1500 ms; TE, 1.9 ms; FA, 9°; matrix, 256×232; FOV, 220×250; section thickness,1 mm; and NEX, 1. The parameters of the transverse T2-weighted imaging were as follows: TR, 5160 ms; TE, 91 ms; FA, 124–130°; matrix, 640×510–580; FOV, 175–199×220; section thickness, 5 mm; and NEX, 3.

The transverse DSC perfusion MR imaging was obtained with single-shot gradient-echo echo-planar sequences during the intravenous administration of gadobutrol at a concentration of 0.1 mmol/kg of body weight at a rate of 4 mL/sec using a power injector (Spectris; Medrad, Pittsburgh, PA). A 30-mL bolus injection of saline followed at the same injection rate. For each section, 60 images were acquired at intervals equal to the TR. The parameters were as follows: TR, 1500 ms; TE, 30 ms; FA, 90°; matrix, 128×128; section thickness, 5–6 mm; intersection gap, 1 mm; FOV, 240×240 mm; sections, 15–20; voxel size, 1.875×1.875×5 mm^3^; pixel bandwidth, 1563 Hz; and total acquisition time, 1 minute 30 seconds.

### Image Postprocessing

DSC perfusion MR images were processed by use of two MR perfusion analysis methods, manual and semiautomatic segmentation, using commercialized software (Nordic ICE, and Nordic TumorEx, NordicNeuroLab, Bergen, Norway, respectively), in which the CE-T1WI and T2WI were used for structural imaging. The rCBV maps were generated by use of established tracer kinetic models applied to the first-pass data [13], [14]. To reduce the recirculation effects, the ΔR2* (1/T2*) curves were fitted to a gamma-variate function, which is an approximation of the first-pass response as it would appear in the absence of recirculation or leakage. The dynamic curves were mathematically corrected to reduce contrast-agent leakage effects [15]. The rCBV and normalized rCBV (nCBV) maps were presented as color overlays on structural images in the manual and semiautomatic methods, respectively. Coregistration between the structural images and rCBV or nCBV maps (color overlay) was automatically accomplished using mutual information based on an algorithm that facilitated the search for an optimal rigid transformation that aligned the two datasets [4], [16]–[18].

On a pixel-by-pixel basis, the rCBV maps were normalized by dividing each rCBV value in a mass by the mean rCBV value in the selected ROI on contralateral normal-appearing white matter, and the ROI locations were determined by the observers using the manual method. Two observers decided the location (contralateral frontal lobe, e.g., centrum semiovale) through review of other normal brain MRI images prior to the rCBV measurement. The area of the ROI was at least 40 mm^2^. No surrounding gray matter was included within the ROI [3], [9].

The nCBV maps were automatically generated. The mean value of the rCBV values outside the tumor was chosen for normalization by the software.

### Image Analysis

The nCBV values were independently analyzed by two board-certified neuroradiologists (S.C.J. and J.A.Y.). All perfusion and structural images were stored anonymized at the perfusion analysis workstation. Each observer measured the nCBV values using T2WI and CE-T1WI for structural images twice over a 5-month period. In total, 8 time measurements were performed for each mass (e.g., two measurements using T2WI with the manual method, two using the CE-T1WI with the manual method, two using the T2WI with the semiautomatic segmentation, and two using CE-T1WI with the semiautomatic segmentation). Each nCBV measurement was performed with an interval of at least 1 week to reduce recall bias. Observers did not share the characteristics of masses with each other prior to drawing the ROI or volume of interest (VOI) because we wanted the study environment to be similar to a realistic clinical arena. Some differences between the observers in regard to the characteristics of masses could contribute to interobserver disagreement. After measurement, the observers analyzed the characteristics of the masses by consensus. The characteristics were as follows: cyst or necrosis, definite perilesional edema, hemorrhage, intralesional macrovessels, heterogeneity, definition of border, definite mass effect, and crossing of the midline [4], [19].

**Figure 2:**
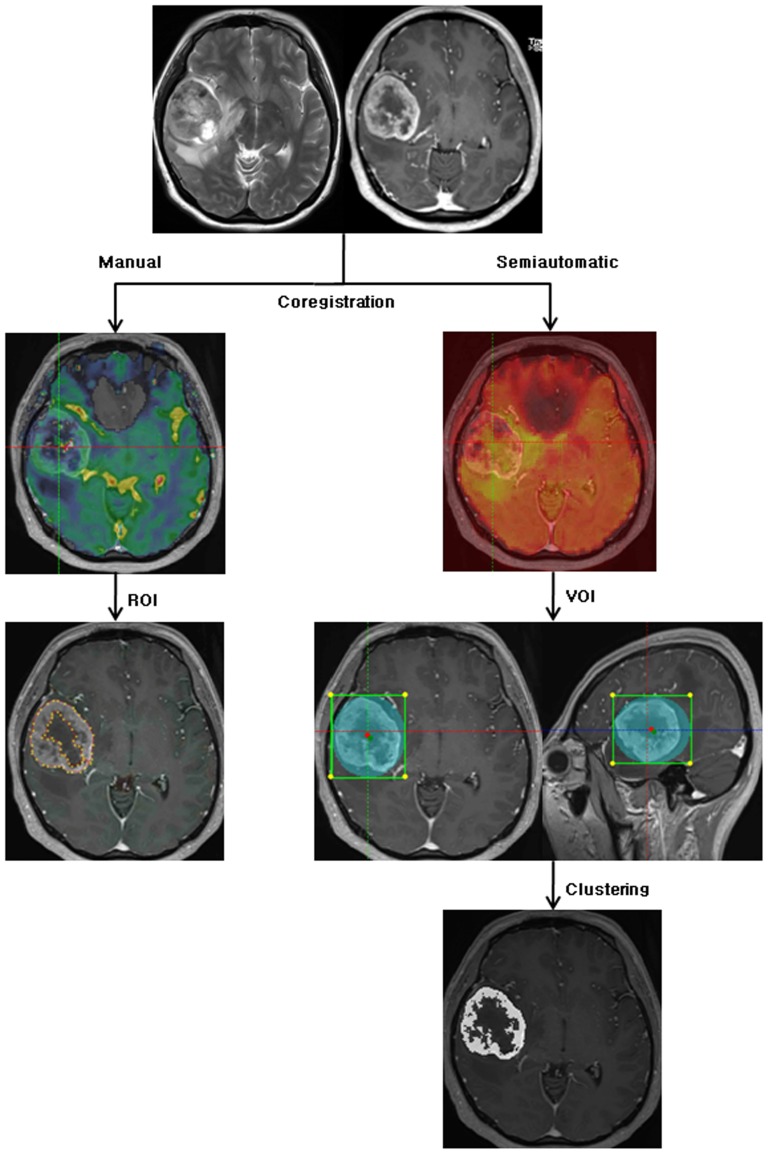
Flowchart of manual and semiautomatic segmentation analysis. Structural imaging (CE-T1WI or T2WI) and nCBV maps were coregistered using the manual segmentation method, and then the ranges of tumors were manually depicted by each observer using an ROI **(right row)**. Structural imaging (CE-T1WI or T2WI) and nCBV maps were coregistered using the semiautomatic segmentation method; then, the ranges of tumors were depicted by each observer using a VOI. Finally, an appropriate combination of clusters from the various clusters was determined by each observer **(left row)**. CE-T1WI = contrast enhanced T1-weighted imaging; T2WI = T2-weighted imaging; nCBV = normalized cerebral blood volume; ROI = region of interest; VOI = volume of interest.

The observers defined a margin of a mass on each axial plane by manually outlining on the T2WI and CE-T1WI using perfusion analysis software (Nordic ICE). All nCBV values were calculated from an outline (ROI) on an axial plane. The total nCBV values in a mass were obtained by averaging the values from every plane. The outline of the mass, ROI, was defined by avoiding the cystic or necrotic regions, intralesional macrovessels, or perilesional edema. We recorded the mean and standard deviation of the nCBV values of each mass according to each structural image (CE-T1WI or T2WI) (Fig. 2). Tumor size was defined as the largest anteroposterior, superoinferior, and transverse dimensions on MR images, and the tumor volumes were calculated using the following formula: volume = 0.5×anteroposterior×superoinferior×transverse dimensions [20], [21]. Cystic or necrotic regions were not included in the tumor volume measurement.

The semiautomatic segmentation was performed using perfusion analysis software (Nordic TumorEx). A VOI was defined by adjusting the elliptical VOI manually on the software and then the automatic segmentation was considered within only the defined VOI. Observers were required to define a mass on the structural imaging that avoided the cystic or necrotic regions, intralesional macrovessels, or perilesional edema. A volume of each mass was also presented because the analysis was performed on the volume data derived from three-dimensional analysis. The automatic segmentation with the clustering analysis was performed after determination of a VOI. The clustering analysis progressed under Expectation and Maximization algorithm. The software could present 3 to 7 clusters, which were not overlapped one another in the segmented VOI. We chose a seven-cluster module within the segmented VOI, and the observers selected some clusters using visual inspection to avoid intralesional cystic and necrotic tissue and macrovessels. The nCBV values from the whole pixels of the selected clusters within the segmented VOI were calculated, and the mean and standard deviation were obtained in each mass according to each structural image (CE-T1WI or T2WI) (Fig. 2). The volume information for each tumor was automatically calculated within the segmented VOI for every tumor.

### Statistical Analysis

Commercially available software (MedCalc, version 11.1.1.0, MedCalc software, Mariakerke, Belgium) was used for the analysis. The Kolmogorov-Smirnov test was used to determine whether values were normally distributed. For all statistical analyses, a two-tailed p value of less than 0.05 was considered indicative of a statistically significant difference.

We assessed intraobserver and interobserver reproducibility by use of the interclass correlation coefficient (ICC), Bland-Altman plot, and coefficient of variation (CV). Intraobserver and interobserver assessment was defined as a comparison between the first and second measurement in the same observer and between measurement from observer 1 and observer 2, respectively. For each observer, the intraobserver reproducibility of the mean nCBV was analyzed according to the segmentation methods or structural imaging technique. Interobserver reproducibility for the nCBV measurement was evaluated according to a measurement order (e.g., comparison of first nCBV measurement of nCBV by observer 1 and 2, as each observer measured the nCBV of a mass twice using the same method), segmentation methods, or structural imaging technique. The mean nCBV values were compared with a paired t-test to assess intra- and interobserver reproducibility.

The ICC values were categorized as follows: <0.40, poor; 0.40–0.59, fair; 0.60–0.74, good; and >0.74, excellent [22]. Intraobserver and interobserver reproducibility were depicted with a Bland-Altman plot. CV values were calculated as follows: the standard deviation was divided by the mean, and the result was presented as a percentage (100×SD/mean) [23]. The volumes of the masses measured by the manual segmentation and semiautomatic segmentation methods were assessed with a paired t-test to assess interobserver reproducibility.

## Results

### Mass Characteristics (8 characteristics)

The masses presented cystic or necrotic regions (n = 24 masses, 89%), definite perilesional edema (n = 21, 78%), hemorrhaging (n = 13, 48%), intralesional macrovessels (n = 19, 70%), heterogeneity (n = 22, 81%), definite mass effects (n = 19, 70%), crossing of the midline (n = 4, 15%), poor circumscribed margins (n = 7, 26%) and well-circumscribed margins (n = 20, 74%).

### The nCBV measurement

The nCBV values are presented in Table 1. There was no significant difference in nCBV values in the intraobserver and interobserver comparison except for the interobserver assessment with the T2WI and semiautomatic segmentation method (p<0.01).

**Table 1:** nCBV values and volumes.

nCBV				
	CE-T1WI		T2WI	
	1st^a^	2nd^a^	1st^a^	2nd^a^
**Manual method**				
Observer 1	4.52±2.36	4.57±2.78	4.84±3.12	4.98±3.11
Observer 2	4.81±4.14	4.84±2.79	4.35±3.12	4.78±2.82
**Semiautomatic segmentation**				
Observer 1	7.93±5.92	7.72±5.80	6.32±4.07^b^	6.44±4.14^b^
Observer 2	7.95±6.04	7.71±5.55	4.51±3.61^b^	4.42±2.93^b^

Volume		
	CE-T1WI	T2WI
**Manual method**		
Observer 1	23.67±25.28 cm^3^ c	27.09±26.18 cm^3^ d
Observer 2	18.27±20.27 cm^3^ c	20.71±22.71 cm^3^ d
**Semiautomatic segmentation**		
Observer 1	16.15±17.49 cm^3^	16.72±15.66 cm^3^
Observer 2	16.20±17.61 cm^3^	15.69±16.38 cm^3^

Note- All data are the means ± standard deviation.

a 1^st^ and 2^nd^ indicate interobserver reproducibility between the first and second measurement, respectively.

b c d There was a statistically significant difference in interobserver measurements (p<0.01).

**Figure 3:**
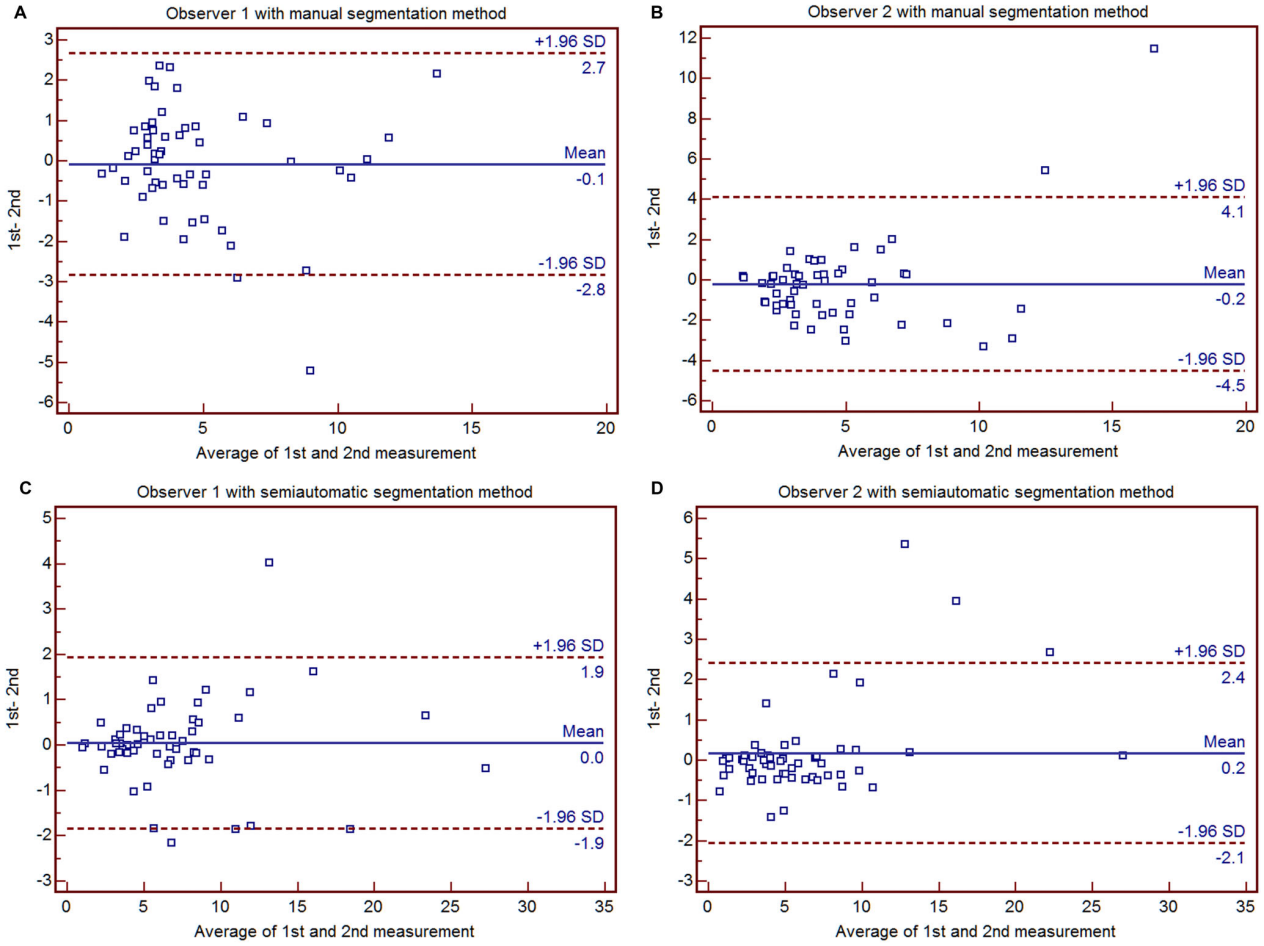
Bland-Altman plots show intraobserver reproducibility between the first and second measurement with observer 1 (a) and observer 2 (b) with the manual method and observer 1 (c) and observer 2 (d) with the semiautomatic segmentation method. Intraobserver reproducibility with the semiautomatic segmentation method was better than that of the manual method for both CE-T1WI- and T2WI-based evaluations. CE-T1WI = contrast enhanced T1-weighted imaging; T2WI = T2-weighted imaging.

Using the manual segmentation method, observer 1 presented an ICC of 0.8660 and a CV of 20.39% with CE-T1WI and an ICC of 0.8864 and a CV of 21.07% with T2WI. Observer 2 presented an ICC of 0.7371 and a CV of 36.83% with CE-T1WI and an ICC of 0.8168 and a CV of 28.18% with T2WI. Using the semiautomatic segmentation method, observer 1 presented an ICC of 0.9872 and a CV of 8.53% with CE-T1WI and an ICC of 0.9714 and a CV of 10.78% with T2WI. Observer 2 presented an ICC of 0.9769 and a CV of 11.26% with CE-T1WI and an ICC of 0.9502 and a CV of 16.19% with T2WI (Table 2). Intraobserver reproducibility using the semiautomatic segmentation method was better than with the manual method for both CE-T1WI- and T2WI-based evaluation according to Bland-Altman plot analysis (Fig. 3).

**Table 2:** Intraobserver reproducibility of nCBV measurement.

	Observer 1		Observer 2	
	Manual method			
	CE-T1WI	T2WI	CE-T1WI	T2WI
**ICC** a	0.87 (0.73–0.94)	0.89 (0.77–0.95)	0.74 (0.50–0.87)	0.82 (0.64–0.91)
**CV** b	20.39	21.07	36.83	28.18

	Semiautomatic segmentation method			
	CE-T1WI	T2WI	CE-T1WI	T2WI
**ICC** a	0.99 (0.97–0.99)	0.97 (0.94–0.99)	0.98 (0.95–0.99)	0.95 (0.89–0.98)
**CV** b	8.53	10.78	11.26	16.19

Note- All numbers in brackets indicate the 95% confidence interval.

a ICC values were categorized as follows: <0.40, poor; 0.40–0.59, fair; 0.60–0.74, good; and>0.74, excellent.

b Numbers are expressed as percentages.

**Figure 4:**
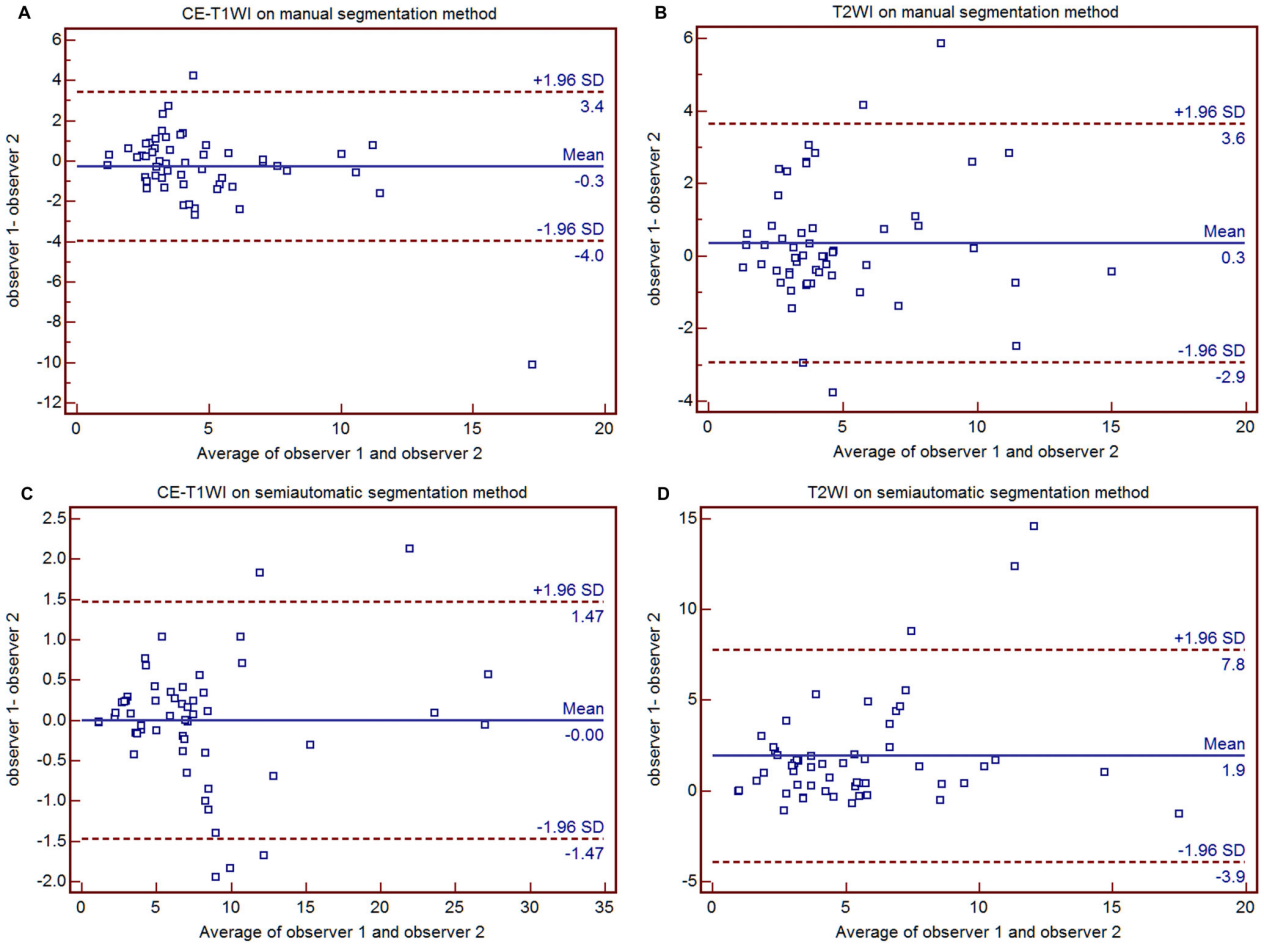
Bland-Altman plots show interobserver reproducibility according to structural imaging technique for both CE-T1WI (a) and T2WI (b) with the manual method and CE-T1WI (c) and T2WI (d) with the semiautomatic segmentation method. Interobserver reproducibility using the semiautomatic segmentation method was better than with the manual method for CE-T1WI-based evaluation and lower than with the manual method for T2WI-based evaluation. CE-T1WI = contrast enhanced T1-weighted imaging; T2WI = T2-weighted imaging.

Using the manual segmentation method, the first measurement produced an ICC of 0.8624 and a CV of 35.15% with CE-T1WI and an ICC of 0.9460 and a CV of 22.64% with T2WI. The second measurement produced an ICC of 0.9420 and a CV of 19.67% with CE-T1WI and an ICC of 0.8826 and a CV of 27.50% with T2WI. Using the semiautomatic segmentation method, the first measurement produced an ICC of 0.9973 and a CV of 5.48% with CE-T1WI and an ICC of 0.8558 and a CV of 42.22% with T2WI. The second measurement produced an ICC of 0.9941 and a CV of 7.81% with CE-T1WI and an ICC of 0.7383 and a CV of 49.38% with T2WI (Table 3). Interobserver reproducibility using the semiautomatic segmentation method was better than with the manual method for CE-T1WI-based evaluation and lower than with the manual method for T2WI-based evaluation in ICC and CV (Table 3) and Bland-Altman plot analysis (Fig. 4).

**Table 3:** Interobserver reproducibility of nCBV measurement.

	Manual method			
	1st^a^ CE-T1WI	1st^a^ T2WI	2nd^a^ CE-T1WI	2nd^a^ T2WI
**ICC** b	0.86 (0.70–0.94)	0.95 (0.88–0.98)	0.94 (0.87–0.97)	0.88 (0.74–0.95)
**CV** c	35.15	22.64	19.67	27.5

	Semiautomatic segmentation method			
	1st^a^ CE-T1WI	1st^a^ T2WI	2nd^a^ CE-T1WI	2nd^a^ T2WI
**ICC** b	1.0 (0.99–1.0)	0.86 (0.68–0.93)	0.99 (0.99–1.0)	0.74 (0.43–0.88)
**CV** c	5.48	42.22	7.81	49.38

Note- All numbers in brackets indicate the 95% confidence interval.

a 1^st^ and 2^nd^ indicate interobserver reproducibility between the first and second measurement, respectively.

b ICC values were categorized as follows: <0.40, poor; 0.40–0.59, fair; 0.60–0.74, good; and>0.74, excellent.

c Numbers are expressed as percentages.

### Tumor volume measurement

The volumes of the masses are presented in Table 1. There were significant differences in the interobserver comparisons of CE-T1WI and T2WI (p<0.01). The interobserver ICC and CV were, respectively, 0.9728 and 30.65% for CE-T1WI and 0.9740 and 29.50% for T2WI (Table 4).

**Table 4:** Interobserver reproducibility of the mass volumes.

Manual method		
	CE-T1WI	T2WI
**ICC** a	0.97 (0.89–0.98)	0.95 (0.89–0.98)
**CV** b	30.65	29.50

Semiautomatic segmentation method		
	CE-T1WI	T2WI
**ICC** a	0.99 (0.97–1.0)	0.93 (0.84–0.97)
**CV** b	16.67	36.84

Note- All numbers in brackets indicate the 95% confidence interval.

a ICC values were categorized as follows: <0.40, poor; 0.40–0.59, fair; 0.60–0.74, good; and>0.74, excellent.

b Numbers are expressed as percentages.

The volumes of masses are presented in Table 1. There was no significant difference according to the interobserver comparisons of CE-T1WI and T2WI (p>0.05). The interobserver ICC and CV were, respectively, 0.9880 and 16.67% for CE-T1WI and 0.9266 and 36.84% for T2WI (Table 4).

## Discussion

Our study provides a direct comparison of manual and semiautomatic segmentation methods used to assess the nCBV of glioblastomas. The main findings of the present study follow. (a) The semiautomatic segmentation method shows higher intraobserver reproducibility than the manual method for both CE-T1WI- and T2WI-based evaluations. (b) Higher interobserver reproducibly was observed using the semiautomatic segmentation method than when using the manual method only for CE-T1WI-based evaluations. (c) The semiautomatic segmentation method provided higher intraobserver and interobserver reproducibility for CE-T1WI-based evaluations than for T2WI-based evaluations. (d) In terms of tumor volume measurement, the semiautomatic method based on CE-T1WI also revealed the highest reproducibility of any evaluated method.

Previous reports have used the manual segmentation method for the evaluation of intracranial tumors with various parameters [4], [7], [9], [24]. Wetzel et al. [3] studied the comparison of three methods for measuring rCBV based on DSC perfusion MR imaging and mentioned the reproducibility of rCBV measurements as follows: an intraobserver ICC of 0.55–0.81 and CV of 31–43% and an interobserver ICC of 0.69–0.71 and CV of 30–43%. They concluded that the reproducibility of rCBV measurements based on manual segmentation was clinically acceptable. Emblem et al [4] also reported moderate (κ = 0.559) and almost perfect (κ = 0.923) interobserver agreement of rCBV measurement using DSC perfusion MRI in which the tumors were also manually segmented. The manual segmentation results of our study presented an intraobserver ICC of 0.74–0.89 and a CV of 20.39–36.83% and an interobserver ICC of 0.86–0.95 and CV of 19.67–35.15%, which was similar to previous studies. In addition, we found that the reproducibility with the semiautomatic segmentation method was superior to the manual segmentation method reproducibility except for T2WI-based evaluations. There was an intraobserver ICC of 0.95–0.99 and CV of 8.53–19.19% and an interobserver ICC of 0.74–0.1.0 and CV of 7.81–49.38%. Bland-Altman plots were demonstrated narrower 95% limits of agreement in higher ICC or CV groups (Fig. 3c and 4c). In contrast, Bland-Altman plots showed wider 95% limits of agreement in lower ICC or CV groups (Fig. 3b and 4d). With respect to the semiautomatic segmentation method, the perfusion maps were automatically generated, whereas each observer had to determine the location and ROI area in the contralateral normal-appearing white matter for normalization in the manual segmentation method, which can possibly result in a lack of reproducibility in perfusion map generation. Automatic tumor segmentation has been demonstrated to have some advantages, including better reproducibility, time efficiency, and standardized criteria for tumor characterization [25], [26]. In this study, the clustering method was used for tumor segmentation. Clustering analysis works under the assumptions that vectors belonging to pixels within a specific type of tissue are similar and tissue classes can be described with a Gaussian distribution. A vector consisted of corresponding pixel values. In the clustering analysis, pixels were allocated to the class with the highest probability of finding the vector in a tissue class as determined by an Expectation-Maximization algorithm. In other words, each cluster could be defined as an assemblage of similar perfusion values. Three to seven clusters were presented in a VOI containing a mass. In this study, a seven-cluster was chosen, and an appropriate combination of clusters reflecting true tumor tissue was determined by each observer. This combination of clusters could easily exclude an intralesional cyst or necrosis and macrovessels compared to the manual segmentation method. Thus, we believe that the semiautomatic segmentation method presented higher intraobserver and interobserver reproducibility than the manual method.

We found lower reproducibility for T2WI-based evaluations than for CE-T1WI-based evaluations using the semiautomatic segmentation method. In addition, the interobsever reproducibility for T2WI in the semiautomatic segmentation method was lower than in manual segmentation method. In the semiautomatic segmentation method, the segmentation consisted of defining VOI manually and the automatic segmentation under the algorithm. The automatic segmentation with the clustering analysis proceeded under the Expectation-Maximization algorithm after defining the VOI. The algorithm may have a limitation in modeling because of overlapping T2 signal intensities between normal tissue and brain tumors [27]. Automatic segmentation algorithms including Expectation-Maximization algorithm are still challenging work although efficient and reliable studies were developed, particularly in non-enhancing T2WI [27]–[30]. In addition it is well known that T2WI provides relatively similar T1 and T2 relaxation parameters between pathology, edema, and scar tissue comparing with CE-T1WI [30]. Although the semiautomatic segmentation tool provides the clusters of signal intensities on T2WI, the signal intensity differences among the clusters seem to be not enough to separate the several tumor components including the solid, cystic or necrotic tissue and the hemorrhagic regions and peritumoral changes such as edema and microscopic tumor infiltration [29], [31].

We found that the tumor volumes measured by the semiautomatic segmentation method were smaller than the manual segmentation volumes for both T2WI- and CE-T1WI-based evaluations, and the semiautomatic segmentation method based on CE-T1WI had a higher reproducibility than the other methods. We believe that the semiautomatic segmentation method can provide the reliable exclusion of cystic or necrotic regions within the tumors, which contributes to smaller volumes of masses as well as higher reproducibility of tumor volume measurement compared with the manual segmentation method. As a result, the nCBV values measured by semiautomatic segmentation method were also higher than the values determined by the manual segmentation method.

This study had some limitations. First, we exclusively included glioblastomas, which allow for good discrimination from surrounding normal brain tissue, while low-grade gliomas have a poorly defined margin [32], [33]. However, we believe that glioblastomas provided benefits for the testing of reproducibility, as they allow for the exclusion of confounding factors such as discordant tumor margin and focus on the influence of each perfusion method or structural imaging technique. Second, observers measured the nCBV values of one mass a total of eight times over a five-month period with an interval of at least one week between each measurement. Although we tried to reduce recall bias, the possibility of bias cannot be completely excluded. However, in the case of interobserver measurement, there was no definite association between reproducibility and measurement order. Thus, we do not believe that this limitation resulted in serious bias. Third, we chose nCBV as the parameter to evaluate reproducibility among the many perfusion-related parameters. Thus, future studies involving other perfusion parameters are warranted.

In conclusion, the semiautomatic segmentation method based on CE-T1WI had the highest intraobserver and interobserver reproducibility compared to other methods for the nCBV measurement of glioblastomas, even though both the manual and semiautomatic segmentation methods were clinically acceptable for nCBV measurement with structural imaging. We expect our results to contribute to future perfusion analysis studies.
